# IUC24427-84 Reducing UTI and sepsis after flexible cystoscopy: impact of a protocol-based approach in high-risk patients—outcomes from a high-volume university teaching hospital

**DOI:** 10.1093/oncolo/oyaf248.026

**Published:** 2025-08-29

**Authors:** L Gradwell, A Pietropaolo

**Affiliations:** University Hospital Southampton, United Kingdom; University Hospital Southampton, United Kingdom

## Abstract

**Background:**

Flexible cystoscopy is a common urological procedure, with a low risk of urinary tract infection and sepsis. This risk is greater in high-risk patients. While guidelines don’t recommend antibiotic prophylaxis, we developed a protocol for high-risk patients undergoing flexible cystoscopy in conjunction with the microbiology department (Figure 1). This was done to counter the increasing number of patients who developed urinary tract infection (UTI) and or sepsis following the procedure.

**Methods:**

In our university hospital, we performed 2,083 and 2,527 flexible cystoscopies in 2023 and 2024, respectively, under local anesthesia (LA) as a day case procedure. We compare the incidence of UTI and sepsis since introducing a protocol-based antibiotic prophylaxis in high-risk patients. This protocol included staff training, reinforcement of aseptic non-touch technique, new sterilizing solution, attention to patient risk factors and antibiotic use when clinically indicated. Data were compared for a 12-month period between 2023 and 2024 (pre- and post-protocol).

**Results:**

UTI rates fell significantly from 0.67% (*n* = 14) to 0.23% (*n* = 6) (*P* < .05), and sepsis requiring ICU admission from 0.24% (*n* = 5) to 0.08% (*n* = 2) (*P* < .04). Notably, 5 of the 6 UTIs in 2024 occurred in patients who met criteria for prophylaxis but did not receive it due to protocol lapses. Adjusting for this, the projected UTI rate would have been 0.04% (*n* = 1) (*P* = .0004).

**Conclusions:**

A structured, protocol-driven approach significantly reduced infectious complications after flexible cystoscopy. However, non-adherence contributed to avoidable infections. We plan to reinforce this protocol through mandatory staff induction, visible reminders in clinical areas, and regular training updates.

